# Reorganization of the Medical College of Ohio

**Published:** 1837

**Authors:** 


### Art. III.—Reorganization of the Medical College of Ohio.

We congratulate the public on the successful reorganization of this valuable "state institution." The following gentlemen compose its new and permanent faculty:

1. John T. Shotwell, A. B. et M. D., *Probationary* professor of Anatomy and Physiology.

2. John Locke, M. D., professor of Chemistry and Pharmacy;—if he should not, on his return from Europe, accept the Chair to which he has been called in Louisville.

3. John Moorhead, M. D., professor of Obstetrics and the Diseases of Women and Children;—his *valedictory* course, as he intends to resign, at the end of the next session, and return to Ireland.

4. M. Búrr Wright, M. D., professor of Materia Medica;—he still residing in Columbus, and continuing as physician to the convicts in the penitentiary.

5. J. P. Kirtland, M. D., professor of the Theory and Practice of Medicine;—provided, we suppose, he will renounce the study of conchology, resign the office of assistant geologist of Ohio, refrain from sitting in the Legislature,resume the practice of physic, and, lastly, accept the professorship.

6. R. D. Mussey, M. D., *Lecturer* on Surgery, for *one* session, to enter on his duties by the first of February, at *farthest.*

7. J. Delamater, M. D., *Substitute* for Dr. Mussey, through the month of January.

Having, but a few months since, lost no less than two-thirds of its professors, by their preference for other institutions, it could not have been expected, that the utmost efforts of those who remained behind, could, in so short a time, have effected such an excellent reorganization; and the result can, perhaps, only be explained on the theory, that *alma mater* has been so long in the *habit* of *reorganizing*, that she has at last acquired uncommon aptitude in that function. D.

